# Improvement of growth performance of *Amorpha fruticosa* under contrasting regime of water and fertilizer in coal-contaminated spoils using response surface methodology

**DOI:** 10.1186/s12870-020-02397-1

**Published:** 2020-04-25

**Authors:** Rana Roy, Mohammad Golam Mostofa, Jinxin Wang, Ashim Sikdar, Tanwne Sarker

**Affiliations:** 1 College of Natural Resources and Environment, Northwest A&F University, Yangling, Shaanxi 712100 People’s Republic of China; 2 Department of Agroforestry & Environmental Science, Sylhet Agricultural University, Sylhet, 3100 Bangladesh; 3 Department of Biochemistry and Molecular Biology, Bangabandhu Sheikh Mujibur Rahman Agricultural University, Gazipur, 1706 Bangladesh; 4 Key Laboratory of Plant Nutrition and the Agri-Environment in Northwest China, Ministry of Agriculture, Yangling, 712100 People’s Republic of China; 5 School of Economics and Finance, Xi’an Jiaotong University, Xi’an, 710049 People’s Republic of China

**Keywords:** *Amorpha fruticosa*, Central composite design, Coal-mined spoil, Growth performance, Nutrients, Revegetation, Soil-water

## Abstract

**Background:**

Water availability and nutrient-status of soils play crucial roles in seedling establishment and plant survival in coal-spoiled areas worldwide. Restoration of spoils pertains to the application of proper doses of nutrients and water, and selection of particular plant species for efficient revegetation. This study aimed at examining the potential effects of different combinations of soil-water and fertilizers (nitrogen, N and phosphorus, P) on morpho-physiological and biochemical attributes of *Amorpha fruticosa* grown in coal-mined spoils. Three factors five-level central-composite-design with optimization technique response surface methodology (rsm) was used to optimize water irrigation and fertilizer application strategies.

**Results:**

Our results revealed a strong correlation between experimental data and predicted values developed from the rsm model. The best responses of *A. fruticosa* in terms of plant height, stem diameter, root length, and dry biomass were observed under a high-water regime. Low-water regime caused a notable reduction in growth-associated parameters, and fertilization with either N or P did not show positive effects on those parameters, indicating that soil-water was the most influential factor for growth performance. Leaf water potential, gas-exchange parameters, and chlorophyll content significantly increased under high levels of soil-water, N and P, suggesting a synergistic effect of these factors for the improvement of photosynthesis-related parameters. At low soil-water contents and N-P fertilizer application levels, enhanced accumulation of malondialdehyde and proline indicated that *A. fruticosa* suffered from oxidative and osmotic stresses. *Amorpha fruticosa* also responded to oxidative stress by accelerating the activities of superoxide dismutase, catalase, and peroxidase. The effects of both fertilizers relied on soil-water, and fertilization was most effective under well-watered conditions. The maximum growth of *A. fruticosa* was observed under the combination of soil-water, N-dose and P-dose at 76% field capacity, 52.0 mg kg^− 1^ and 49.0 mg kg^− 1^, respectively.

**Conclusion:**

Our results demonstrate that rsm effectively designed appropriate doses of water and N-P fertilizer to restore coal-spoiled soils. Furthermore, *A. fruticosa* responded to low-water and fertilizer-shortage by upregulating defensive mechanism to avoid damage induced by such deficiencies. Finally, our findings provide effective strategies for revegetation of coal-contaminated spoils with *A. fruticosa* using appropriate doses of water and N-P fertilizers.

## Background

Water-shortage is considered as one of the major ecological limiting factors, affecting the restoration of vegetation and plant productivity. Simultaneously, the soil-water deficiency is usually connected with the unobtainability of nutrients in arid and semi-arid areas of the world [1]. Drought along with nutrient deficiency limits vegetation establishment, and the relationships between vegetation restoration and soil-moisture availability have recently gathered significant attention because of their importance in re-establishment of an ecosystem in the drought-prone areas [2–4]. The northwest-part of China considerably covers a big region of the world’s dry-lands [5]. Seedling establishment and plant growth in this region largely depend on natural precipitation [5]. Moreover, this region comprises of many coal-mine areas, contributing around 70% of China’s coal production. Coal-mining leads to serious ecological and environmental problems, such as removal of the earth-surface cover, damage of plant roots, destruction of vegetation and turning of coal-mining areas into deserts [6, 7]. Thus, the coal-mined spoils of northwest-part of China could be an appropriate resource to explore the possibility of ecosystem restoration using particular plant species and appropriate doses of water and fertilizers.

Artificial ecological engineering (revegetation) is the appropriate method for restoration of ecology in an abandoned coal gob piles in order to improve the environmental conditions. However, restoration of such kind of degraded areas is very challenging and considered as a daunting task for the environmentalists [8]. An adequate amount of soil-moisture and nutrients is the key to support plant growth and development in dry-land areas [9]. Moreover, the development of a self-sustaining revegetation ecosystem also depends on the selection of suitable plant species, which, together with effective treatment strategies using soil-water and fertilizers can help increase restoration effectiveness [10]. The plants which are originated in the desert-environment have better adaptable photosynthetic systems compared to plants that originated in other normal environments. To continue life cycles in the desert areas, plants develop several survival mechanisms associated with morphological, physiological and biochemical adaptations [11, 12]. *Amorpha fruticosa* is a deciduous shrub (1–6 m tall) and able to grow in diverse environmental conditions [13]. It is a commonly used shrub for revegetation in Loess Plateau of China because of its drought resistance and adaptation capabilities under infertile conditions. *A. fruticosa* is frequently cultivated in areas after landslides and escarpments, and for stabilization of the soils on railway embankments, as it’s roots play important roles in the reduction of soil erosion. It can also effectively dwells in degraded-environments in the form of brushes with its seed and suckers [14].

Ecological problems due to coal mining and the vegetation reestablishment in coal-mining areas are very important environmental issues, urging in-depth research for the sustainable environment [8, 15]. Numerous findings reported the impacts of soil-water and nutrients on the growth and development of various crops in coal-mined areas [16–18]. The results suggested that the coupling effects of soil-water and fertilizers played vital roles on plant growth for the restoration of vegetation. To our knowledge, the combinatorial effects of soil-water and fertilizers on desert shrubs in coal-mined spoils have rarely been performed. Hence, it is worth investigating the growth response of *A. fruticosa* planted in coal-mined spoils concerning different strategies of water and fertilizer applications. Thus, the objective of this study was to evaluate the growth and development of *A. fruticosa* in response to different combination of water (W) and fertilizers (nitrogen, N and phosphorus, P) using central composite design (CCD) with optimization technique response surface methodology (rsm), and to find out optimum water and fertilizer doses for vegetation restoration in a coal-contaminated desert area in China. For this purpose, we have examined the effect of W, N and P on the morphological, physiological and biochemical responses of *A. fruticosa* by considering plant height, stem diameter, root length, dry biomass, root/shoot (R/S) ratio of biomass, leaf water potential (LWP), photosynthesis rate (*P*_n_)*,* transpiration rate (*T*_r_), stomatal conductance (*G*_s_), water use efficiency (WUE), the levels of chlorophyll (Chl), malondialdehyde (MDA), and proline (Pro), and the activities of antioxidant enzymes superoxide dismutase (SOD), catalase (CAT) and peroxidase (POD).

## Results

### Effects on morphological growth responses of *A. fruticosa*

The maximum increase of plant height (101.23 cm), stem diameter (7.26 mm), root length (73.12 cm) and dry biomass (42.37 g) were recorded under the treatments of W (80% Field capacity (FC)), N (60 mg kg^− 1^) and P (90 mg kg^− 1^) (Table 1). The minimum root-shoot (R/S) biomass ratio of 1.51 was observed when the plants were treated with the highest dose of W (80% FC), and medium doses of N (60 mg kg^− 1^) and P (90 mg kg^− 1^) fertilizers (Table 1). The lowest increment of plant height (52.04 cm) was recorded under the combination of W, N and P at 48.1% FC, 95.68 mg kg^− 1^ and 36.49 mg kg^− 1^, respectively (Table 1). The increase of stem diameter was 4.83 mm when the plants exposed to medium W (60% FC) and N (60 mg kg^− 1^), and highest P doses (180 mg kg^− 1^) (Table 1). On the other hand, the lowest root length (44.96 cm) was recorded when the plants were treated with medium W (60% FC), N (60 mg kg^− 1^) and P (90 mg kg^− 1^) doses (Table 1). The minimum dry biomass (24.0 g) and highest R/S biomass ratio (2.09) were observed at W (40% FC), N (60 mg kg^− 1^) and P (90 mg kg^− 1^) doses (Table 1). Positive values of the parameter coefficients W (a_1_) and N (a_2_) exhibited positive effect of W, and N on plant height, stem diameter, and root length, and the relative magnitude was: W > N (Table 3). In contrast, P (a_3_) had no positive effect on the above-mentioned parameters (Table 3). For plant dry biomass parameter coefficients, W, N, and P had a positive effect with W having the greatest effect, followed by N and P (Table 3). The coefficient P was positive for R/S biomass ratio in contrast W and N had a negative effect (Table 3).

**Table 1:** Effect of different combination of soil-water (W), nitrogen (N) and phosphorus (P) on plant height, stem diameter, root length, dry biomass, root-shoot (R/S) biomass ratio, photosynthesis rate (*P*_n_), transpiration rate (*T*_r_), stomatal conductance (*G*_s_) and water use efficiency (WUE) of *A. fruticosa*

No. of Trt	Level of treatments			Plant height (cm)	Stem diameter (mm)	Root length (cm)	Dry biomass (g)	R/S biomass ratio	LWP (−MPa)	*P*_*n*_ (μmol m^− 2^ s^− 1^)	*T*_r_ (mmol m^− 2^ s^− 1^)	*G*_s_ (mol m^− 2^ s^− 1^)	WUE (μmol mmol^− 1^)
	W (% FC)	N (mg kg^− 1^)	P (mg kg^− 1^)										
1	48.1	24.32	36.49	64.12 ± 12.2^def^	5.23 ± 0.22^fg^	50.33 ± 3^bcd^	24.01 ± 2.72^e^	2.03 ± 0.04^ab^	4.21 ± 0.08^j^	11.13 ± 0.37^d^	4.47 ± 0.06^ef^	0.23 ± 0.005^ef^	2.49 ± 0.05^m^
2	71.9	24.32	36.49	88.25 ± 4.67^ab^	6.43 ± 0.41^bc^	52.84 ± 3.15^bcd^	34.25 ± 2.17^bc^	1.61 ± 0.02^gh^	1.92 ± 0.02^d^	12.34 ± 0.37^c^	3.77 ± 0.05^hi^	0.22 ± 0.005^fg^	3.27 ± 0.05^b^
3	48.1	95.68	36.49	52.04 ± 7.1^f^	5.26 ± 0.26^efg^	54.95 ± 3.87^bcd^	24.84 ± 2.38^e^	1.8 ± 0.02^e^	3.83 ± 0.08^i^	12.62 ± 0.23^bc^	4.06 ± 0.09^g^	0.22 ± 0.002^fg^	3.11 ± 0.03^c^
4	71.9	95.68	36.49	86.5 ± 5.6^abc^	6.73 ± 0.22^ab^	59.46 ± 2.42^b^	35.97 ± 3.08^b^	1.56 ± 0.03^hi^	1.63 ± 0.04^c^	12.57 ± 0.32^bc^	4.71 ± 0.08^de^	0.23 ± 0.006^ef^	2.67 ± 0.02^hi^
5	48.1	24.32	143.51	58.03 ± 3.21^ef^	4.85 ± 0.19^g^	50.68 ± 5.3^bcd^	25.01 ± 0.83^de^	1.94 ± 0.03^c^	4.16 ± 0.04^j^	10.78 ± 0.16^d^	3.98 ± 0.05^gh^	0.17 ± 0.002^j^	2.71 ± 0.02^gh^
6	71.9	24.32	143.51	71.08 ± 5.89^cde^	5.96 ± 0.26^bcdef^	60.66 ± 1.56^b^	35.48 ± 2.94^b^	1.58 ± 0.03^hi^	1.95 ± 0.04^d^	12.83 ± 0.24^abc^	4.73 ± 0.1^cd^	0.21 ± 0.003^gh^	2.71 ± 0.01^gh^
7	48.1	95.68	143.51	67.78 ± 3.24^de^	5.12 ± 0.1^g^	45.1 ± 3.62^d^	25.83 ± 1.48^de^	1.96 ± 0.02^bc^	3.63 ± 0.05^h^	12.68 ± 0.28^bc^	3.64 ± 0.0^6i^	0.19 ± 0.004^i^	3.48 ± 0.01^a^
8	71.9	95.68	143.51	85.5 ± 5.95^bc^	6.52 ± 0.29^abc^	59.82 ± 2.37^b^	36.66 ± 3.51^ab^	1.68 ± 0.02^fg^	1.42 ± 0.03^b^	13.52 ± 0.34^a^	5.9 ± 0.13^a^	0.26 ± 0.005^bc^	2.29 ± 0.01^n^
9	40	60	90	64.06 ± 4.04^def^	5.11 ± 0.48^g^	58.48 ± 5.92^bc^	24.01 ± 1.21^e^	2.09 ± 0.02^a^	4.81 ± 0.05^k^	10.92 ± 0.24^d^	3.73 ± 0.05^hi^	0.22 ± 0.004^fg^	2.93 ± 0.04^de^
10	80	60	90	101.23 ± 8.69^a^	7.26 ± 0.46^a^	73.12 ± 8.52^a^	42.38 ± 2.93^a^	1.51 ± 0.04^i^	1.07 ± 0.09^a^	13.02 ± 0.26^abc^	5.05 ± 0.11^b^	0.25 ± 0.004^cd^	2.58 ± 0.01^jkl^
11	60	0	90	64 ± 2.16^def^	5.45 ± 0.19^defg^	47.21 ± 2.36^d^	28.85 ± 1.61^cde^	1.72 ± 0.01^f^	3.22 ± 0.04^g^	11.24 ± 0.27^d^	3.93 ± 0.09^gh^	0.21 ± 0.004^gh^	2.86 ± 0.02^ef^
12	60	120	90	71.1 ± 2.58^cde^	6.04 ± 0.36^bcde^	50.35 ± 2.15^bcd^	30.88 ± 1.41^bcd^	1.69 ± 0.02^fg^	2.52 ± 0.04^e^	13.32 ± 0.28^ab^	4.43 ± 0.09^f^	0.24 ± 0.004^de^	3.01 ± 0.02^d^
13	60	60	0	71 ± 3.48^cde^	5.47 ± 0.14^defg^	51.51 ± 2.5^bcd^	27.02 ± 0.88^de^	1.94 ± 0.01^c^	2.87 ± 0.04^f^	12.3 ± 0.26^c^	4.41 ± 0.11^f^	0.21 ± 0.004^gh^	2.79 ± 0.02^fg^
14	60	60	180	63.3 ± 4.4^def^	4.83 ± 0.1^g^	51.15 ± 3.88^bcd^	28.52 ± 1.61^cde^	1.86 ± 0.02^de^	2.67 ± 0.06^e^	12.54 ± 0.22^bc^	4.73 ± 0.05^cd^	0.2 ± 0.002^hi^	2.65 ± 0.02^hij^
15	60	60	90	74.04 ± 5.05^bcd^	5.92 ± 0.39^cdef^	47.53 ± 2.52^d^	29.27 ± 1.59^cde^	1.93 ± 0.03^cd^	3.03 ± 0.05^f^	12.3 ± 0.28^c^	4.77 ± 0.1^cd^	0.27 ± 0.004^ab^	2.58 ± 0.02^ijkl^
16	60	60	90	75.37 ± 4.14^bcd^	5.98 ± 0.2^bcdef^	46.22 ± 3.18^d^	28.96 ± 2.13^cde^	1.95 ± 0.01^c^	2.93 ± 0.06^f^	12.34 ± 0.42^c^	4.86 ± 0.09^bcd^	0.28 ± 0.007^a^	2.54 ± 0.04^klm^
17	60	60	90	73.34 ± 3.22^bcde^	6.12 ± 0.16^bcd^	44.96 ± 1.97^d^	29.38 ± 1.61^cde^	1.94 ± 0.02^cd^	2.95 ± 0.05^f^	12.42 ± 0.26^c^	4.76 ± 0.06^cd^	0.27 ± 0.002^ab^	2.61 ± 0.02^ijkl^
18	60	60	90	71.02 ± 5.75^cde^	5.96 ± 0.12^bcdef^	48.18 ± 2.3^cd^	29.35 ± 1.4^cde^	1.95 ± 0.03^bc^	3.02 ± 0.04^f^	12.54 ± 0.25^bc^	4.98 ± 0.13^bc^	0.26 ± 0.003^bc^	2.52 ± 0.03^lm^
19	60	60	90	72.67 ± 5.35^bcde^	6.05 ± 0.23^bcd^	45.65 ± 3.92^d^	29.51 ± 1.78^cde^	1.9 ± 0.02^cd^	3 ± 0.07^f^	12.48 ± 0.34^c^	4.75 ± 0.09^cd^	0.28 ± 0.005^a^	2.63 ± 0.02^hijk^
20	60	60	90	73.52 ± 3.95^bcde^	5.98 ± 0.18^bcdef^	47.78 ± 4.06^cd^	29.01 ± 1.78^cde^	1.95 ± 0.03^c^	2.96 ± 0.03^f^	12.52 ± 0.26^bc^	4.72 ± 0.08^cde^	0.26 ± 0.006^bc^	2.65 ± 0.01^hij^

Values are means ± standard errors (*n* = 4). Different alphabetical letters (a, b, c, etc) within the same column indicate significant differences among various treatments according to a least significant difference test (LSD) (*P* <  0.05). *Trt* treatment, *FC* field capacity

### Effects on leaf water potential, gas-exchange parameters, and chlorophyll contents of *A. fruticosa*

The maximum increase and decrease of leaf water potential (LWP) (− 1.08 MPa and − 4.81 MPa, respectively) were obtained at medium N (60 mg kg^− 1^) and P (90 mg kg^− 1^), but at highest and lowest W (80 and 40% FC) level, respectively (Table 1). The maximum values obtained for photosynthesis rate (*P*_n_) and transpiration rate (*T*_r_) were 13.52 (μmol m^− 2^ s^− 1^) and 5.90 (mmol m^− 2^ s^− 1^), respectively, under the treatments of W at 71.9% FC, N at 95.68 mg kg^− 1^ and P at 143.51 mg kg^− 1^ (Table 1). In contrast, the lowest value of *P*_n_ (10.78 μmol m^− 2^ s^− 1^) and (*G*_s_) (0.17 mol m^− 2^ s^− 1^) were recorded under the combinations of W, N, and P (48.1% FC, 24.32 mg kg^− 1^ and 143.51 mg kg^− 1^, respectively) (Table 1). The treatment with W (48.1% FC), N (95.68 mg kg^− 1^) and P (143.51 mg kg^− 1^) resulted in minimum *T*_r_ (3.64 mmol m^− 2^ s^− 1^) and maximum water use efficiency (WUE) (3.48 μmol mmol^− 1^) (Table 1). All coefficients for LWP, *P*_n_, and *T*_r_ were positive (Table 3). Although W and N positively influenced *G*_s_, WUE was effectively increased by N only. For *G*_s_ and WUE*,* P did not show any positive effect (Table 3).

The minimum levels of chlorophyll (Chl) *a* (2.16 mg g^− 1^ fresh weight (FW)), Chl *b* (1.06 mg g^− 1^ FW) and total Chls (3.22 mg g^− 1^ FW) were observed in response to the treatments with W at 60% FC, N at 0 mg kg^− 1^ and P at 90 mg kg^− 1^ (Table 2). The highest levels of Chl *a* (2.69 mg g^− 1^ FW) and total Chl (4.69 mg g^− 1^ FW) were obtained in response to the treatments with W at 80% FC, N at 60 mg kg^− 1^ and P at 90 mg kg^− 1^ (Table 2). Chl *a*, Chl *b* and total Chl contents in *A. fruticosa* leaves significantly increased by the addition of W, N, and P, where N had the maximum positive impact followed by W and P (Table 3).

**Table 2:** Effect of different combination of soil-water (W), nitrogen (N) and phosphorus (P) on the levels of chlorophyll a (Chl *a*), chlorophyll b (Chl *b*), total chlorophylls (Total Chls), malondialdehyde (MDA) and proline (Pro), and the activities of superoxide dismutase (SOD), catalase (CAT) and peroxidase (POD) in the leaves of *A. fruticosa*

No. of Trt	Level of treatments			Chl *a* (mg g^− 1^ FW)	Chl *b* (mg g^− 1^ FW)	Total Chls (mg g^− 1^ FW)	MDA (μmol g^− 1^ FW)	Pro (μmol g^− 1^ FW)	SOD (U g^− 1^ FW)	CAT (U min^− 1^ g^− 1^ FW)	POD (U min^− 1^ g^− 1^ FW)
	W (% FC)	N (mg kg^− 1^)	P (mg kg^− 1^)								
1	48.1	24.32	36.49	2.38 ± 0.04^e^	1.49 ± 0.04^fg^	3.87 ± 0.03^gh^	35.23 ± 4.59^ab^	3.96 ± 0.03^c^	139.56 ± 6.98^abc^	193.21 ± 3.93^ab^	213.74 ± 5.58^b^
2	71.9	24.32	36.49	2.54 ± 0.01^b^	1.78 ± 0.06^cd^	4.32 ± 0.05^de^	33.42 ± 4.12^abcd^	2.12 ± 0.03^l^	117.45 ± 8.12^efgh^	182.34 ± 3.09^bcd^	132.75 ± 5.38^k^
3	48.1	95.68	36.49	2.41 ± 0.07^de^	1.68 ± 0.06^de^	4.09 ± 0.04^fg^	30.74 ± 2.2^abcde^	5.15 ± 0.04^a^	142.31 ± 7.27^ab^	187.78 ± 7.17^abc^	161.21 ± 7.14^ghi^
4	71.9	95.68	36.49	2.52 ± 0.02^bcd^	1.77 ± 0.07^cd^	4.29 ± 0.05^ef^	23.34 ± 2.07^e^	2.56 ± 0.03^j^	107.38 ± 4.28^hijk^	167.48 ± 4.2^efgh^	187.03 ± 7.85^cdef^
5	48.1	24.32	143.51	2.31 ± 0.05^e^	1.32 ± 0.03^g^	3.63 ± 0.07^i^	31.15 ± 4.63^abcde^	2.32 ± 0.04^k^	135.87 ± 10.5^bcd^	173.28 ± 3.43^def^	254.66 ± 8.43^a^
6	71.9	24.32	143.51	2.42 ± 0.04^cde^	1.4 ± 0.05^g^	3.82 ± 0.09^hi^	26.01 ± 2.26^de^	2.93 ± 0.04^hi^	133.24 ± 3.31^bcde^	152.97 ± 3.61^i^	149.42 ± 6.28^ijk^
7	48.1	95.68	143.51	2.59 ± 0.02^ab^	1.97 ± 0.08^ab^	4.56 ± 0.1^abc^	36.74 ± 2.53^a^	3.04 ± 0.03^h^	129.13 ± 5.15^bcdefg^	192.28 ± 5.01^ab^	143.77 ± 5.5^jk^
8	71.9	95.68	143.51	2.61 ± 0.01^ab^	2.02 ± 0.06^a^	4.63 ± 0.08^ab^	26.43 ± 2.65^cde^	2.82 ± 0.05^i^	113.4 ± 5.92^ghij^	168.53 ± 3.27^efgh^	155.45 ± 7.4^hij^
9	40	60	90	2.53 ± 0.04^bc^	1.84 ± 0.03^bcd^	4.37 ± 0.06^cde^	34.41 ± 5.05^abc^	2.88 ± 0.03^i^	154.68 ± 9.42^a^	196.15 ± 3.95^a^	212.68 ± 7.38^b^
10	80	60	90	2.69 ± 0.04^a^	2 ± 0.04^ab^	4.69 ± 0.04^a^	25.81 ± 0.94^de^	1.54 ± 0.03^m^	109.63 ± 3.68^hijk^	171.05 ± 3.79^efg^	148.88 ± 5.32^ijk^
11	60	0	90	2.16 ± 0.07^f^	1.06 ± 0.04^h^	3.22 ± 0.11^j^	31.82 ± 2.17^abcd^	4.27 ± 0.06^b^	131.36 ± 7.31^bcdef^	167.59 ± 2.3^efgh^	201.24 ± 3.31^bc^
12	60	120	90	2.36 ± 0.03^e^	1.59 ± 0.03^ef^	3.95 ± 0.06^gh^	28.48 ± 1.37^abcde^	4.15 ± 0.04^b^	115.05 ± 4.46^fghi^	178.51 ± 5.79^cde^	148.32 ± 4.26^ijk^
13	60	60	0	2.53 ± 0.05^bc^	1.86 ± 0.03^abc^	4.39 ± 0.08^cde^	31.3 ± 3.52^abcde^	3.62 ± 0.02^ef^	118.6 ± 7.85^defgh^	184.76 ± 2.17^bc^	169.93 ± 7.67^fgh^
14	60	60	180	2.55 ± 0.04^b^	1.87 ± 0.02^abc^	4.42 ± 0.06^bcde^	29.28 ± 1.3^abcde^	2.37 ± 0.06^k^	124.27 ± 7.16^cdefgh^	158.86 ± 3.24^hi^	174.63 ± 4.77^efg^
15	60	60	90	2.57 ± 0.02^b^	1.96 ± 0.07^ab^	4.53 ± 0.09^abcd^	29.34 ± 2.58^abcde^	3.73 ± 0.06^de^	96.16 ± 4.09^jkl^	164.34 ± 3.6^fgh^	191.82 ± 3.7^cde^
16	60	60	90	2.56 ± 0.02^b^	1.95 ± 0.02^ab^	4.51 ± 0.02^abcde^	28.15 ± 1.82^bcde^	3.81 ± 0.06^d^	94.32 ± 3.03^kl^	166.73 ± 2.55^fgh^	187.03 ± 7.03^cdef^
17	60	60	90	2.58 ± 0.01^ab^	1.94 ± 0.08^abc^	4.52 ± 0.09^abcd^	29.56 ± 3.68^abcde^	3.62 ± 0.04^ef^	97.96 ± 3.78^ijkl^	161.39 ± 3.74^ghi^	183.31 ± 5.45^def^
18	60	60	90	2.55 ± 0.02^b^	1.94 ± 0.1^abc^	4.49 ± 0.11^abcde^	32.33 ± 1.87^abcd^	3.49 ± 0.04^fg^	89.22 ± 3.98^l^	168.92 ± 2.56^efgh^	189.53 ± 6.54^cde^
19	60	60	90	2.56 ± 0.03^b^	1.98 ± 0.07^ab^	4.54 ± 0.09^abcd^	31.06 ± 2.14^abcde^	3.85 ± 0.03^cd^	95.6 ± 2.35^kl^	171.49 ± 3.74^defg^	193.15 ± 4.88^cd^
20	60	60	90	2.54 ± 0.02^b^	1.92 ± 0.08^abc^	4.46 ± 0.09^abcde^	31.15 ± 2.8^abcde^	3.43 ± 0.05^g^	93.27 ± 6.51^kl^	165.97 ± 3.65^fgh^	185.38 ± 5.5^cdef^

Values are means ± standard errors (*n* = 4). Different alphabetical letters (a, b, c, etc) within the same column indicate significant differences among various treatments according to a least significant difference test (LSD) (*P* <  0.05). *Trt* treatment, *FC* field capacity

**Table 3:** Parameter coefficients of eq. (Y = a_0_ + a_1_x_1_ + a_2_x_2_ + a_3_x_3_ + a_4_x_1_x_2_ + a_5_x_1_x_3_ + a_6_x_2_x_3_ + a_7_x_1_^2^ + a_8_x_2_^2^ + a_9_x_3_^2^) for plant height (R_1_), stem diameter (R_2_), root length (R_3_), total biomass (R_4_), Root-shoot biomass ratio (R_5_), leaf water potential (R_6_), photosynthesis rate (R_7_), transpiration rate (R_8_), stomatal conductance (R_9_), water use efficiency (R_10_), chlorophyll a (R_11_), chlorophyll b (R_12_), total chlorophyll (R_13_), malondialdehyde (R_14_), proline (R_15_), superoxide dismutase activity (R_16_), catalase activity (R_17_) and peroxidase activity (R_18_)

Y	a_0_	a_1_	a_2_	a_3_	a_4_	a_5_	a_6_	a_7_	a_8_	a_9_	R^2^	R^2^_*a*_	R^2^_*p*_	F	P	CV	AP
R_1_	73.35	11.12	1.63	−1.57	1.88	−3.48	4.75	3.14	−2.20	−2.34	0.990	0.981	0.950	113.09	< 0.0001	2.11	43.42
R_2_	6.0	0.64	0.16	−0.17	0.07	−0.02	0.06	0.08	− 0.07	− 0.28	0.992	0.985	0.963	147.34	< 0.0001	1.32	46.57
R_3_	46.77	4.13	0.74	−0.14	0.84	2.21	−2.21	6.41	0.39	1.29	0.980	0.963	0.906	55.99	< 0.0001	2.59	26.90
R_4_	29.25	5.39	0.58	0.47	0.15	−0.01	−0.07	1.38	0.20	−0.53	0.999	0.998	0.997	1344.44	< 0.0001	0.63	135.78
R_5_	1.94	−0.17	−0.02	0.001	0.03	0.001	0.05	−0.05	−0.09	− 0.02	0.980	0.963	0.870	56.83	< 0.0001	1.8	24.19
R_6_	−2.99	1.11	0.21	0.06	−0.01	−0.01	0.04	0.02	0.04	0.08	0.999	0.998	0.998	1725.45	< 0.0001	1.16	156.85
R_7_	12.43	0.55	0.57	0.11	−0.31	0.22	0.11	− 0.14	− 0.03	0.02	0.984	0.970	0.910	70.70	< 0.0001	1.04	32.12
R_8_	4.80	0.38	0.16	0.13	0.36	0.38	0.04	−0.14	−0.21	− 0.07	0.987	0.975	0.950	84.14	< 0.0001	1.91	36.77
R_9_	0.27	0.01	0.008	−0.006	0.004	0.01	0.008	−0.01	−0.02	− 0.02	0.976	0.955	0.917	46.15	< 0.0001	2.86	20.73
R_10_	2.59	−0.11	0.05	−0.04	− 0.30	−0.19	0.04	0.06	0.13	0.05	0.990	0.981	0.977	111.66	< 0.0001	1.42	43.35
R_11_	2.56	0.05	0.06	0.008	−0.02	−0.01	0.05	0.02	−0.10	−0.005	0.994	0.990	0.981	213.48	< 0.0001	0.48	62.01
R_12_	1.95	0.06	0.17	0.0005	−0.03	−0.03	0.14	−0.01	−0.22	− 0.03	0.993	0.987	0.955	167.61	< 0.0001	1.68	46.73
R_13_	4.51	0.11	0.23	0.008	−0.05	−0.05	0.19	0.007	−0.33	−0.04	0.996	0.993	0.982	325.74	< 0.0001	0.72	69.39
R_14_	30.29	−2.86	−1.04	−0.42	−1.35	−0.78	2.57				0.939	0.912	0.911	33.75	< 0.0001	3.29	21.55
R_15_	3.65	−0.46	0.15	−0.35	− 0.20	0.60	− 0.13	− 0.51	0.19	− 0.23	0.963	0.929	0.782	28.79	< 0.0001	6.95	22.08
R_16_	94.43	−11.07	−4.49	1.06	−3.24	4.84	−2.41	13.29	10.13	9.51	0.985	0.972	0.927	73.19	< 0.0001	2.74	24.82
R_17_	166.46	−8.59	2.39	−6.39	−1.61	−1.61	6.86	6.16	2.43	1.99	0.963	0.930	0.853	28.85	< 0.0001	1.85	20.05
R_18_	188.01	−18.75	−14.07	1.21	27.97	−4.79	−13.33	−2.65	−4.77	−5.66	0.993	0.987	0.973	161.49	< 0.0001	1.86	51.97

### Effects on malondialdehyde and proline accumulation, and the activities of antioxidant enzymes of *A. fruticosa*

The malondialdehyde (MDA) content of *A. fruticosa* seedlings was suppressed by all 3 factors; however, proline (Pro) content was considerably decreased by W and P (Table 3). Among the factors, W was most influential for decreasing MDA and Pro contents in the leaves of *A. fruticosa* (Table 3). The minimum MDA content of 23.34 μmol g^− 1^ FW was recorded with W at 71.9% FC, N at 95.68 mg kg^− 1^ and P at 36.49 mg kg^− 1^ (Table 2). In contrast, the maximum MDA content was observed under the combinations of W, N, and P (48.1% FC, 95.68 mg kg^− 1^ and 143.51 mg kg^− 1^, respectively) (Table 2). The highest W (80% FC) and medium N (60 mg kg^− 1^) and P (90 mg kg^− 1^) doses represented minimum accumulation of Pro (1.54 μmol g^− 1^ FW) in the leaves of experimental seedlings; while the maximum Pro content of 5.15 μmol g^− 1^ FW was obtained with W at 48.1% FC, N at 95.68 mg kg^− 1^ and P at 36.49 mg kg^− 1^ (Table 2).

The superoxide dismutase (SOD) (154.68 U g^− 1^ FW) and catalase (CAT) (196.15 U min^− 1^ g^− 1^ FW) activities considerably increased under the treatment with W at 40% FC, N at 60 mg kg^− 1^ and P at 90 mg kg^− 1^. The minimum SOD activity (89.22 U g^− 1^ FW) was observed at medium W (60% FC), N (60 mg kg^− 1^), and P (90 mg kg^− 1^) levels, whereas the minimum CAT activity (152.97 U min^− 1^ g^− 1^ FW) was recoreded with W at 71.9% FC, N at 24.32 mg kg^− 1^ and P at 143.51 mg kg^− 1^. The lowest peroxidase (POD) activity (132.75 U min^− 1^ g^− 1^ FW) in the leaves of *A. fruticosa* was observed with the combinations of W, N, and P (71.9% FC, 95.68 mg kg^− 1^ and 36.49 mg kg^− 1^, respectively). However, the POD activity significantly increased (254.66 U min^− 1^ g^− 1^ FW) in response to the treatments with W at 48.1% FC, N at 95.68 mg kg^− 1^ and P at 143.51 mg kg^− 1^ (Table 2). The activities of SOD and POD were decreased by W and N; while CAT activity was considerably decreased by W and P (Table 3).

### Interactive effect of process variables on morphological parameters

**Fig. 1:**
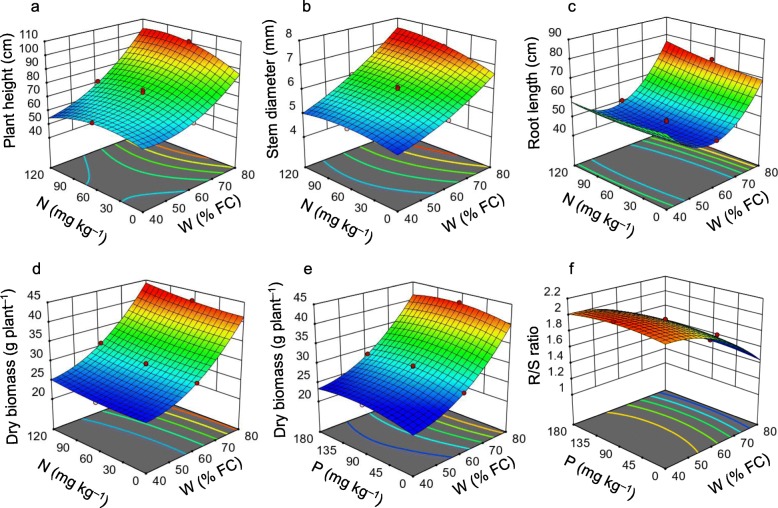
Response surface plots showing the effect of soil-water (W), nitrogen (N) and phosphorus (P) on the plant height (**a**), stem diameter (**b**), root length (**c**), dry biomass (**d**-**e**), and root-shoot (R/S) biomass ratio (**f**)

The mutual effect of W, N, and P over plant height and stem diameter displayed that plant height and stem diameter increased linearly with W rising from 40 to 80% FC. At low W dose (40% FC), increment of N and P doses showed a little positive effect on the elevation of plant height and stem diameter, whereas at high W dose (80% FC), plant height and stem diameter significantly increased with the addition of N and P fertilizers (Fig. 1a, b, Additional file 1: Figure S1). The root length initially decreased with the decrease of W dose (from 80 to 60% FC), but after reaching a certain point (at 60% FC), it started increasing with the further reduction of W dose (Fig. 1c, Additional file 1: Figure S1). Under low W dose (40% FC), N fertilizer did not express any significant effects, while at high W dose (80% FC) addition of N fertilizer increased root length slowly (Fig. 1c, Additional file 1: Figure S1). Dry biomass of *A. fruticosa* seedlings significantly influenced in response to W dose regardless of the doses of N and P fertilizers. The positive influence of N on plant dry biomass was detected at a high W dose (80% FC) (Fig. 1d). In contrast, under low (40% FC) and high (80% FC) W doses, the addition of P-fertilizer slightly increased dry biomass, but continuous P-addition resulted in negative impacts (Fig. 1e). The R/S biomass ratio dramatically enhanced with the decline of W level, whereas P had no obvious effects on R/S biomass ratio (Fig. 1f, Additional file 1: Figure S1).

### Interactive effect of process variables on LWP, gas-exchange parameters, and chlorophyll contents

**Fig. 2:**
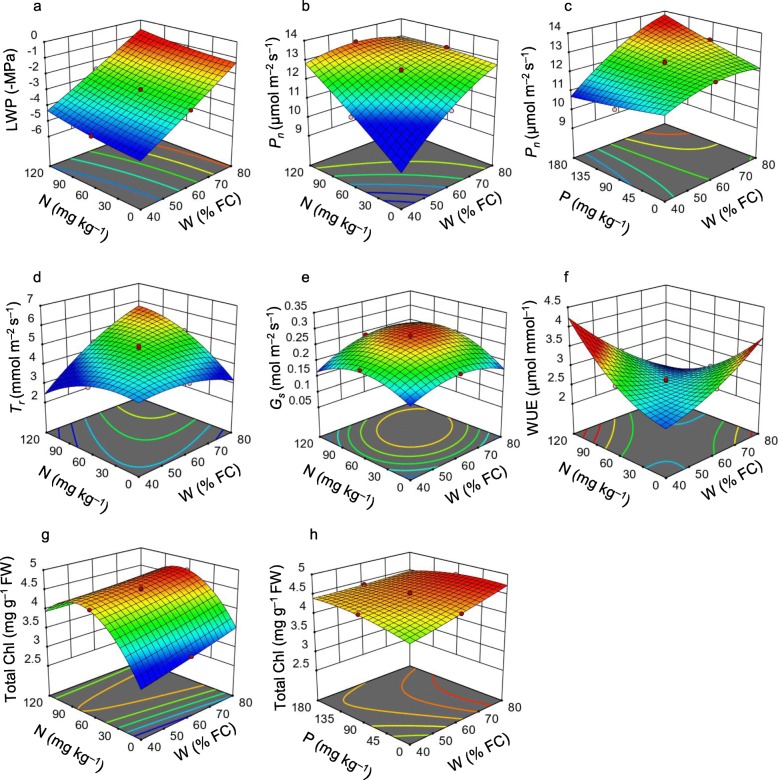
Response surface plots showing the effect of soil-water (W), nitrogen (N) and phosphorus (P) on the leaf water potential (LWP) (**a**), photosynthesis rate (*P*_*n*_) (**b**, **c**), transpiration rate (*T*_r_) (**d**), stomatal conductance (*G*_s_) (**e**), water use efficiency (WUE) (**f**), and total chlorophyll (total Chl) content (**g**-**h**)

It was observed that LWP significantly declined at low W condition (around 40 to 48.1% FC), and the addition of N and P fertilizers did not show any positive effects on the recovery of LWP (Fig. 2a, Additional file 2: Figure S2). A mutual effect of W with N-P fertilizers over *P*_n_ and *T*_r_ were observed, and the data showed that both *P*_n_ and *T*_r_ increased with the addition of W, N and P, and the maximum *P*_n_ and *T*_r_ were obtained at W (71.9% FC), N (95.68 mg kg^− 1^) and P (143.51 mg kg^− 1^) doses (Fig. 2b-d, Additional file 2: Figure S2). The *G*_s_ initially increased with W and N doses up to a certain level (W at 60% FC and N at 60 mg kg^− 1^), whereas it started to decline with the increase of W and N above this level (Fig. 2e). Under low W condition (48.1% FC), WUE significantly increased with the increase of N (95.68 mg kg^− 1^) and P doses (143.51 mg kg^− 1^) (Fig. 1f, Additional file 2: Figure S2). The combined effect of W and N, and W and P on Chl *a*, Chl *b,* and total Chls indicated that under both low and high W levels, total Chl contents dramatically increased with the addition of N, whereas the interactive effect of W and P had no significant influence on the levels of total Chl (Fig. 2g-h, Additional file 2: Figure S2).

### Interactive effect of process variables on MDA and pro contents, and antioxidant enzyme activities

**Fig. 3:**
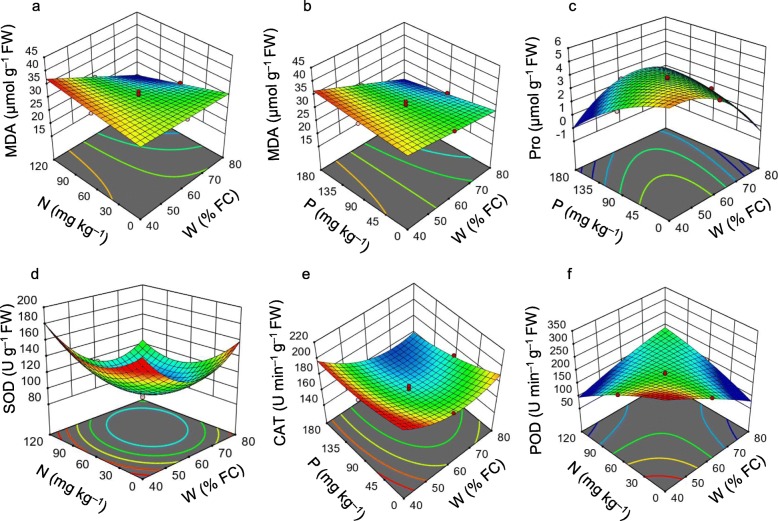
Response surface plots showing the effect of soil-water (W), nitrogen (N) and phosphorus (P) on the malondialdehyde (MDA) (**a**-**b**) and proline (Pro) (**c**) contents, and activities of superoxide dismutase (SOD) (**d**), catalase (CAT) (**e**), and peroxidase (POD) (**f**)

Under low W condition (48.1% FC), the addition of N (95.68 mg kg^− 1^) and P (143.51 mg kg^− 1^) fertilizer dramatically increased MDA content in our experimental seedlings (Fig. 3a-b, Additional file 3: Figure S3). The highest W level (80% FC) and medium N-P fertilizer (60 mg kg^− 1^ and 90 mg kg^− 1^, respectively) content was beneficial for the decrease of Pro content (Fig. 3c, Additional file 3: Figure S3).

The medium dose of W (60% FC) and N-P fertilizer (60 mg kg^− 1^ and 90 mg kg^− 1^, respectively) was suitable for the decrease of SOD activity (Fig. 3d, Additional file 3: Figure S3). When W level was 71.90% FC, CAT activity significantly decreased with the increase of P fertilizer (Fig. 3e, Additional file 3: Figure S3). Under low W and N doses (48.1% FC and 24.32 mg kg^− 1^, respectively), POD activity considerably increased. On the other hand, POD activity exhibited a decreasing trend in response to the increase of either W or N-P fertilizers (Fig. 3f, Additional file 3: Figure S3).

### Optimization of soil-water, nitrogen and phosphorus fertilizer

**Fig. 4:**
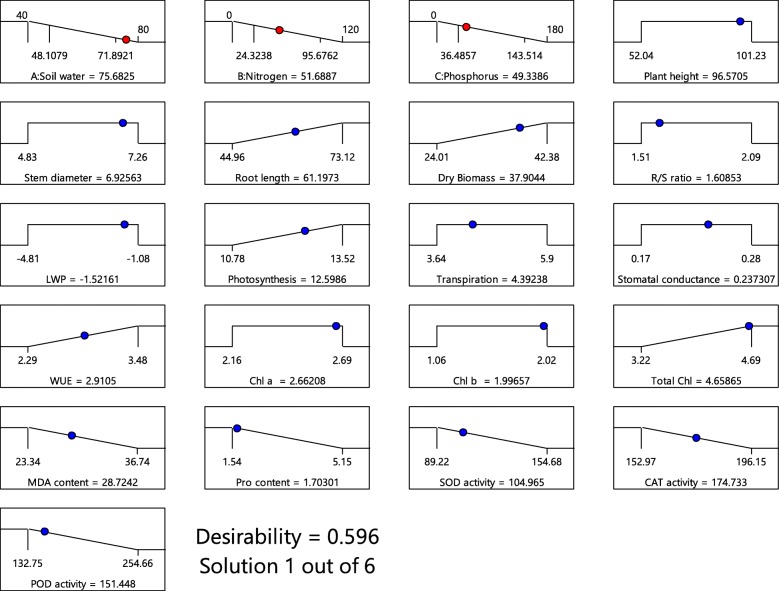
Desirability ramp for optimization

According to CCD result, the optimum water and fertilizer rate to obtain maximum growth performance of *A. fruticosa* was determined by Derringer’s desired function approach as follows: W of 75.68% FC, N of 51.64 mg kg^− 1^, P of 49.49 mg kg^− 1^ (Fig. 4). Under this condition, maximum plant growth was obtained (Fig. 4). Accurateness of the optimized condition was identified by comparing the average experimental values and the predicted values developed by the model. The desirability ramp obtained from optimal points is represented in Fig. 4.

## Discussion

Our study demonstrated that maximum plant height, stem diameter, root length, and dry biomass were obtained at high soil-water content; and in this situation, the addition of N-P fertilizers significantly improved these growth associated attributes (Fig. 1a-e, Additional file 1: Figure S1). Low water levels caused a significant decline in plant dry biomass, and the effect of N-P fertilizers also depended on the availability of soil-water. At low water-level, increases in the doses of N-P fertilizers exhibited a little effect on the improvement of plant biomass, and the highest biomass was obtained at high water level (Fig. 1d-e; Additional file 1: Figure S1; Table 1). These findings indicated that due to scarcity of soil-water, nutrients present in the soil did not mobilize well, and plant roots could not uptake it adequately, which ultimately prevented water and nutrients flow through the xylem to the surrounding cells, as observed in other plant species under water-shortage conditions [19–21]. Thus, the deficiency of nutrients and water eventually led to the inhibition of cell growth and elongation [21], as was also manifested in this study in terms of plant height and stem diameter (Table 1). However, the mutual influence of soil-water and N-P fertilizers considerably improved the functions of each other, which ultimately positively increased the plant height, stem diameter, root length and dry biomass of *A. fruticosa*, which were in parallel with previous studies [22, 23]. The previous study reported that soil-water was the most important factor for plant morphological responses, and the effect of fertilizers like N and P on plant growth depends on soil-water content [24]. In this experiment, we observed that R/S biomass ratio significantly increased under low water regime (Fig. 1f; Additional file 1: Figure S1; Table 1), indicating that under water and nutrient shortage conditions, carbon translocated from leaves to roots, thereby increasing R/S biomass ratio [25]. The increased R/S biomass ratio during stress conditions could also be explained as one of the adaptive mechanisms of plants to make greater root surfaces for absorbing much water and nutrients from soils [26]. However, other study reported that a greater R/S biomass ratio under stress conditions was resulted due to decreased shoot growth [27].

LWP acts as a powerful indicator of plants’ water status, and a reduced level of water has been known to be involved in declining LWP in plant species [28]. Similarly, we observed that low level of water caused a severe reduction of LWP in *A. fruticosa* (Fig. 2a; Additional file 2: Figure S2; Table 1). Gas-exchange parameters are considered as important physiological parameters, having significant roles in plant growth and development. The status of gas-exchange parameters also correlated with the adaptive responses of the plants to cope under environmental assaults [3]. We also observed that interaction of high level of water with high-level fertilizers significantly increased *P*_n_, *T*_r_, and *G*_s_, whereas water and fertilizer limitations led to a considerable decline of those gas-exchange parameters (Fig. 2b-f; Additional file 2: Figure S2; Table 1). Under adverse environmental conditions, stomata of leaves are closed and *P*_n_*is* inhibited, and thus, declined levels of *G*_s_ and *P*_n_ have been considered as indications of stomatal limitation [29]. Furthermore, a noticeable decrease in *G*_s_ during stress conditions indicate an efficient adaptive mechanism to control *T*_r_ [30]. The severe water-shortage condition is extremely unfavourable for absorbing and utilizing nutritional components from the soils by the plant root-systems [31]. Under well-watered conditions, a reasonable coupling model of water and fertilizers could be adopted to utilize N and P [32]. Our results corroborated with the findings of several reports, and indicated that high soil-water content and fertilizers positively increased *P*_n_, *G*_s_ and *T*_r_; however, plants are used to reduce these parameters in order to survive under water-shortage conditions [28, 33]. Our experimental results exhibited that during water-shortage conditions, WUE increased with the addition of N-P fertilizer, and the minimum WUE was detected under high soil-water and N-P fertilizer doses, which were in parallel with a previous report [34].

The status of Chl content is considered as the tolerance index of the plants under stressful conditions [35]. Total Chl (Chl *a* + Chl *b*) content considerably decreased with the increasing level of water-shortage (Fig. 2g-h; Additional file 2: Figure S2; Table 2), indicating that *A. fruticosa* mounted an adaptive mechanism to protect themselves from the damage of low-water induced photo-inhibition [36]. Gallé and Feller [37] also reported that continuous drought led to decrease Chl content, reduce stomatal conductance, and finally reduce *P*_n_ in the leaves of *Fagus sylvatica*. However, together with N, soil-water significantly increased Chl content, whereas P did not show any positive effect on Chl content. Since N is an integral part of Chl molecules, N-shortage conditions were associated with the reduced level of Chl and chlorosis-related symptoms in plants [38, 39].

We observed a considerable increase of lipid peroxidation product MDA and osmoprotectant Pro under water-shortage condition compared to the well-watered conditions (Fig. 3a-c; Additional file 3: Figure S3; Table 2). These results suggest that water deficiency led to the production of ROS, which ultimately caused lipid peroxidation of the membrane, leading to greater production of MDA. However, MDA content decreased gradually with the addition of soil-water and N-P fertilizer, and the results showed that high W and N-P fertilizer doses contributed to drought tolerance by inhibiting the damage of cell membranes and enhancing osmoregulation in *A. fruticosa* seedlings. Saneoka et al. [40] also reported that MDA content in bentgrass (*Agrostis palustris*) was higher under drought conditions than those found in control condition, and MDA content decreased with the addition of N to the growth medium. Similarly, a water-deficient state resulted in osmotic stress, and *A. fruticosa* accumulated a high level of Pro to overcome the adverse effects of osmotic stress. Our results corroborated the findings of others in mulberry (*Morus alba*) and moth beans (*Vigna aconitifolia*) [41, 42], and suggested that water-stressed plants increased osmoprotective adaptation by accumulating high levels of osmoprotectants like Pro.

To protect from the deleterious effects of ROS produced under stressful conditions, plants normally increase their protective mechanisms against ROS by enhancing the activities of several enzymes, including SOD, CAT and POD [11]. A wealth of reports suggest that exposure of plants to drought led to the enhancement of the activities of antioxidant enzymes, such as SOD, CAT, and POD, and increased enzymatic responses represent a greater antioxidant capacity for providing drought resistance in plants [43–45]. A remarkable increase in SOD activity in the leaves of low W and high N-treated seedlings (Fig. 3d; Additional file 3: Figure S3; Table 2) indicated that *A. fruticosa* plants improved their antioxidative defence status to protect themselves from ROS-mediated damage. A significant rise in CAT and POD activities in the leaves of *A. fruticosa* plants under low W, N, and P treatments also implied that *A. fruticosa* plants responded well to water and nutrient shortage by activating antioxidant capacity (Fig. 3e-f; Additional file 3: Figure S3; Table 2).

The maximum growth performance of *A. fruticosa* was observed in response to the treatments with W at 75.68% FC, N at 51.64 mg kg^− 1^ and P at 49.49 mg kg^− 1^ (Fig. 4). In accordance with our results, Wang et al. [46] also demonstrated that microbial inoculum with inorganic fertilizer (N at 61.73 mg kg^− 1^ and P at 40.0 mg kg^− 1^ doses) significantly improved morphological, physiological, and antioxidant enzyme responses in *Medicago sativa* when grown in coal-mined spoils.

## Conclusion

The levels of doses developed for W, N and P using rsm correlated with the growth performance of *A. fruticosa*, suggesting that rsm could be used as an effective tool to design the perfect combination of water and fertilizers for revegetation of coal-spoiled soils. Our results revealed that application of various doses of W, N and P significantly altered growth-associated attributes, including shoot height, stem diameter, root length and dry biomass of *A. fruticosa*. Although all the three factors (W, N and P) exhibited significant impacts on morphological, physiological and biochemical traits of *A. fruticosa,* W was more prominent in influencing those traits when compared with N and P. The maximum effects on growth-contributing parameters were observed when high level of W (76% FC) was applied with 52.0 mg kg^− 1^ of N and 49.0 mg kg^− 1^ of P. This combination of W, N and P was also highly effective in enhancing the levels of LWP, gas exchange parameters and photosynthetic pigment contents. However, under low doses of W, which might have induced drought stress, *A. fruticosa* enhanced the accumulation of Pro and the activities of antioxidant enzymes SOD, POD and CAT. These results indicated that *A. fruticosa* employed osmoprotective effects of Pro to overcome osmotic stress and antioxidative roles of SOD, CAT and POD to reduce oxidative stress in order to survive under water-shortage conditions. Collectively, the findings of this study provided valuable and useful information that could be used as effective measures for revegetation and ecological restoration of coal-contaminated subsidence areas in China, and perhaps in other arid and semi-arid areas of the world. However, field studies with a range of plant species using suggested doses of W, N and P (76% FC, 52.0 mg kg^− 1^, 49.0 mg kg^− 1^, respectively) would be beneficial to ensure improved management and sustainable restoration of coal-spoiled areas.

## Methods

### Plant material and growth conditions

The coal-mined spoils with topsoil were collected from the Yangchangwan coal mining area (106° 35′ ~ 106° 38′E, 37° 59′ ~ 38° 03′N) of Lingwu city, Ningdong, Ningxia province, China. The sample was naturally dried, mixed, and finally sieved to 5 mm. Fourteen (14) kg of soil was taken to fill up each pot (upper diameter of 32 cm, bottom diameter of 27 cm, and height of 30 cm). One-year-old *A. fruticosa* seedlings with similar height (33.0 ± 3.0 cm) and stem diameter (2.8 ± 0.3 mm) were collected from Hengwang Seedlings Greening Company, Alashan County, Inner Mongolia, China. The plant species were identified based on its characteristics and was approved by the Jilantai Forestry Bureau, Alashan county, Inner Mongolia, China before collection. The collection of coal-mined spoils with topsoil and plant specimens were done in a public right of way, which means no specific permission was required for collecting soil and plant samples, and we have not deposited any voucher specimen. After transplanting one *A. fruticosa* seedling to each pot in March 2018, all pots were placed in an artificial shed at Northwest A&F University, (N 34° 16′, E 108° 4′), Yangling. The mean annual precipitation and temperature of the experimental site (outside of the artificial shed) were 650 mm and 13.7 °C, respectively. In the first month, sufficient water was provided to all pots to induce seedling recovery from transplantation stress. One month later, when the water content was close to the lower level of the water threshold, experimental treatments were applied, up to October 2018 according to the experimental design obtained from rsm. The amount of moisture lost from each pot through transpiration and evaporation was determined through a weighing method, and irrigation was done every day during the entire trial period. Fertilizer was applied in holes near the root zone of the seedlings. Nitrogen (N) fertilizer (urea-46% N) was applied in four times (¼ N, May 6^th^; ¼ N, June 6^th^; ¼ N, July 6^th^; ¼ N, August 6^th^), whereas phosphorus (P) fertilizer (Triple super phosphate-46% P_2_O_5_) was applied in two equal half (^**1**^**/**_**2**_ May 6^th^ and ^**1**^**/**_**2**_ July 6^th^). Before starting the pot experiment, various soil properties were calculated, and recorded as FC (11.177%), soil bulk density (1.48 g cm^− 3^), soil pH (7.83), total N (0.157 g kg^− 1^), available N (6.14 mg kg^− 1^), available P (1.98 mg kg^− 1^) and available K (66.88 mg kg^− 1^).

### Measurement of morphological growth

Changes in plant height and stem diameter were measured using measuring-tape and slide calliper, respectively, at every 15-days-interval throughout the growing seasons. Increase of growth in plant height and stem diameter were calculated by subtracting initial reading from final reading. For the measurement of plant biomass, plant samples were carefully washed with water for removing debris, and different plant parts (leaves, stems, and roots) were separated accordingly. Plant samples were initially dried in an oven for 20 min at 105 °C, followed by further drying at 80 °C, and finally dry biomass of different plant parts was determined. The maximum root length also measured using a measuring-tape. The root-shoot (R/S) biomass ratio was measured by below-ground biomass divided by above-ground biomass.

### Determination of leaf gas-exchange parameters and leaf water potential

Leaf gas-exchange parameters, including net photosynthesis rate (*P*_n_), transpiration rate (*T*_r_), stomatal conductance (*G*_s_) and water use efficiency (WUE) were measured on sunny days between 8:30 am and 11:30 am using a Portable Photosynthesis System CIRAS-3 (PP Systems, Amesbury, MA, U.S.A). Three seedlings from each treatment were used for determining gas-exchange parameters. At the time of measurement, photosynthetic active radiations were maintained at 1000 μmol m^− 2^ s^− 1^, CO_2_ at 300 μmol mol^− 1^, leaf temperature at 25 °c and relative humidity at 70% (provided by a built-in red LED light source). Leaf water potential (LWP) of the selected seedlings was determined by PMS-Model 1000 plant water potential meter (PMS Instrument Company, Albany, OR, USA) before 06:00 am.

### Estimation of chlorophyll contents

Chlorophyll (Chl) was extracted from completely expanded fresh leaf samples (0.1 g) using a 10 mL mixture of ethanol, acetone and distilled water (4.5:4.5:1) following the method of Chen and Chen [47]. The absorbance of the extracts was taken at 645 and 663 nm, and the contents of Chl *a*, Chl *b* and total Chl were calculated as mg g^− 1^ fresh weight (FW) by the formula described by Arnon [48].

### Measurement of malondialdehyde and proline contents

The method of Wang et al. [49] was adopted to extract, and determine the contents of malondialdehyde (MDA) in leaf samples of *A. fruticosa*. Extract (2 mL) was mixed with an equal volume of 0.5% 2-thiobarbituric acid (TBA, dissolved in 15% trichloroacetic acid), and the mixture was heated for 30 min at 100 °C followed by cooling in an ice bath. The mixture was then centrifuged at 10,000 rpm for 10 min, and the absorbance of the supernatants was read at 450, 532 and 600 nm using a spectrophotometer. The MDA content was calculated and expressed as μmol g^− 1^ FW.

Free proline (Pro) from fresh leaf samples was extracted using sulphosalicylic acid solution, and measured with ninhydrin according to Bates et al. [50]. The fresh leaf samples (0.1 g) were extracted with 3% aqueous sulfosalicylic acid (10 mL) and placed in water-bath for boiling for 10 min followed by filtering with Whatman No. 2 filter paper. Plant extract (2 mL) was mixed with 2 mL ninhydrin solution and 2 mL glacial acetic acid, and the mixture was heated for 30 min at 100 °C, followed by cooling in an ice bath. Toluene (4 mL) was used to extract proline from the mixture; the content of Pro was measured after taking the absorbance at 520 nm using a spectrophotometer.

### Determination of antioxidant enzyme activities

Fresh leaf samples (0.3 g) were homogenized in a mortar and pestle (ice-cold conditions) using 8 mL of 50 mM sodium phosphate buffer (pH 7.8), and the homogenates were centrifuged at 10,000 rpm for 20 min at 4 °C. The supernatant was separated and used for the estimation of enzyme activities.

The activity of superoxide dismutase (SOD, EC 1.15.1.1) was determined following the method of Zhang et al. [51]. The reaction mixture contained 3 mL of phosphate buffer (pH 7.8), 0.6 mL of 130 mM methionine buffer, 0.6 mL of 750 μM nitroblue tetrazolium buffer, 0.6 mL of 100 μM EDTA-Na buffer, 0.6 mL of 20 μM riboflavin and 0.2 mL of enzyme extract. The photoreduction of nitroblue tetrazolium was determined at 560 nm using a spectrophotometer. One unit (U) of SOD activity was equal to the amount of enzyme needed to produce 50% inhibition of the color reaction.

Catalase (CAT, EC 1.11.1.6) activity was determined following the method reported in Beers and Sizer [52]. The reaction mixture (2.6 mL) contained 100 mM phosphate buffer (pH 7.0), 20 mM H_2_O_2_ and 100 μL enzyme extract. The reduction in H_2_O_2_ was monitored at 240 nm. Enzyme activity was expressed as CAT U min^− 1^ g^− 1^ FW.

Peroxidase (POD, EC 1.11.1.7) activity was carried out using the guaiacol oxidation method described by Ekmekci and Terzioglu [53]. Enzyme extract (20 μL) was mixed with 3 mL of 50 mM phosphate buffer (pH 6.0) having 20.1 mM guaiacol, 12.3 mM H_2_O_2_, and guaiacol oxidation was measured based on absorbance increase at 470 nm for 3 min. Enzyme activity was expressed as units of guaiacol oxidized min^− 1^ g^− 1^ FW.

### Experimental design for statistical analysis and optimization

In our study, response surface methodology (RSM) based on central composite design (CCD) method was used with statistical software design expert (Trial version 11, Stat-Ease Inc., USA). Ranges of independent variables of our study were chosen based on some previous literature [54–56] and presented in Table 4. Different levels (+α, + 1, 0, − 1, − α) of each factor were taken for operating this study. The distances from the centre to axial points are represented by α and calculated by the following equation.

1 $$ \alpha ={\left({2}^{\mathrm{k}}\right)}^{1/4}, $$

**Table 4:** Factor levels used in central composite design (CCD)

Independent variables	Codes	Symbols	Coded and actual values				
			- 1.682	−1	0	+ 1	+ 1.682
Soil-water (% FC)	x_1_	W	40	48.1	60	71.9	80
Nitrogen (N) rate (mg kg^−1^)	x_2_	N	0	24.32	60	95.68	120
Phosphorus (P) rate (mg kg^−1^)	x_3_	P	0	36.49	90	143.51	180

Where k represents the number of factors, and therefore, α value in our study was 1.682. The CCD comprises 20 experimental runs with eight factorial points, six axial points and six replicates at the center points. A total of 80 seedlings subjected to twenty treatments with four repetitions were arranged randomly. Independent variables effects on responses were analyzed by using a second-order polynomial equation:

2 $$ \mathrm{Y}={\mathrm{a}}_0+{\mathrm{a}}_1{\mathrm{x}}_1+{\mathrm{a}}_2{\mathrm{x}}_2+{\mathrm{a}}_3{\mathrm{x}}_3+{\mathrm{a}}_4{\mathrm{x}}_1{\mathrm{x}}_2+{\mathrm{a}}_5{\mathrm{x}}_1{\mathrm{x}}_3+{\mathrm{a}}_6{\mathrm{x}}_2{\mathrm{x}}_3+{\mathrm{a}}_7{{\mathrm{x}}_1}^2+{\mathrm{a}}_8{{\mathrm{x}}_2}^2+{\mathrm{a}}_9{{\mathrm{x}}_3}^2, $$

Where, Y is the response variables; a_0_ is regression constant-coefficient; a_1_, a_2_ and a_3_ are linear terms; a_4_, a_5_, and a_6_ are interaction terms; a_7_, a_8_, and a_9_ determine quadratic terms; x_1_, x_2_, and x_3_ = represents the coded value of W, N, and P.

RSM was carried out to optimize the process variables on the growth and development of *A. fruticosa,* and the experimental results are presented in Tables 1 and 2. The relationship between the response variables and process variables was obtained by applying multiple regression analysis. The final models, in terms of coded factors were represented in Table 3. A Positive and negative sign in front of the terms indicated synergistic and antagonistic effects, respectively. To determine the significance and fitness of the model for response variables, analysis of variances (ANOVA) was carried out. The model *F* and *P*-value were found for different parameters indicated that the model was highly significant. To determine the model precision, the results of the experiment were examined with coefficient of variation (CV), adequate precision (AP) and R^2^ values. In our present experiment, the adjusted R-squared (R^2^_*a*_) value was detected to be very near and a little bit lesser then R^2^, which designated a strong correlation between the experimental and predicted values. For reasonable agreement, the difference between predicted R-squared (R^2^_*p*_) value and R^2^_*a*_ value should be less than 0.2 [57], and we found that all difference values were less than 0.2 which confirmed that the form of the model chosen to explain the relationship between the factors and the response was well-correlated. 3-D response surface plots obtained from statistical software were designed to find out the relationship between process variables and to assess the response variables over process variables. At the time of creating 3-D response surface plot, we retained our third factor as a central level (as our model comprises three factors).

## Supplementary information

**Additional file 1: Figure S1.** Response surface plots showing the effect of soil-water (W), nitrogen (N) and phosphorus (P) on the plant height (a-b), stem diameter (c-d), root length (e-f), dry biomass (g), and root-shoot (R/S) biomass ratio (h-i).

**Additional file 2: Figure S2.** Response surface plots showing the effect of soil-water (W), nitrogen (N) and phosphorus (P) on the leaf water potential (LWP) (a-b), photosynthesis rate (*P*_*n*_) (c), transpiration rate (*T*_r_) (d-e), stomatal conductance (*G*_s_) (f-g), water use efficiency (WUE) (h-i), chlorophyll a (Chl *a*) (j-l), chlorophyll b (Chl *b*) (m-o) and total chlorophyll (Total Chl) content (p).

**Additional file 3: Figure S3.** Response surface plots showing the effect of soil-water (W), nitrogen (N) and phosphorus (P) on the malondialdehyde (MDA) (a) and proline (Pro) (b-c) contents, and activities of superoxide dismutase (SOD) (d-e), catalase (CAT) (f-g), and peroxidase (POD) (h-i).

## Data Availability

The dataset generated and analyzed during the study are available from the corresponding author on reasonable request.

## Abbreviations

W: Soil-water
N: Nitrogen
P: Phosphorus
R/S ratio: Root to shoot biomass ratio
LWP: Leaf water potential
*P*_n_: Photosynthesis rate
*T*_r_: Transpiration rate
*G*_s_: Stomatal conductance
WUE: Water use efficiency
Chl *a*: Chlorophyll a
Chl *b*: Chlorophyll *b*
total Chl: Total Chlorophyll
FW: Fresh weight
SOD: Superoxide dismutase
CAT: Catalase
POD: Peroxidase
MDA: Malondialdehyde
Pro: Proline
CCD: Central composite design
RSM: Response surface methodology
