# Supplementary material for: Improvement of growth performance of Amorpha fruticosa under contrasting regime of water and fertilizer in coal-contaminated spoils using response surface methodology
Source: BMC Plant Biol. 2020 Apr 25;20:181. doi: 10.1186/s12870-020-02397-1 (PMC7183614; doi:10.1186/s12870-020-02397-1)
Supplement: Supplementary file 1 — Additional file 1: Figure S1. Response surface plots showing the effect of soil-water (W), nitrogen (N) and phosphorus (P) on the plant height (a-b), stem diameter (c-d), root length (e-f), dry biomass (g), and root-shoot (R/S) biomass ratio (h-i). [file 12870_2020_2397_MOESM1_ESM.pptx]

## Slide 1
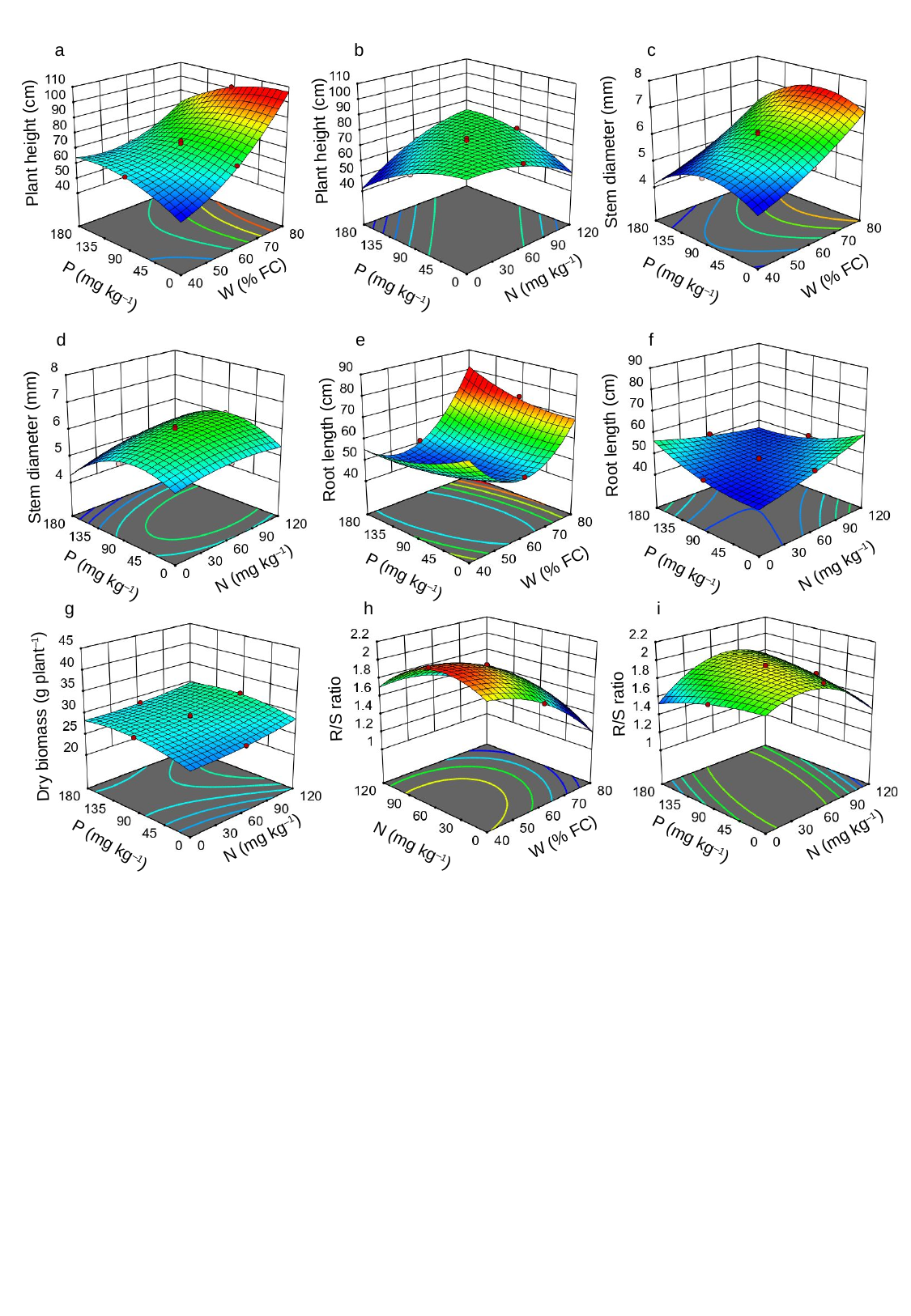

a
b
c
Plant height (cm)
N (mg kg‒1)
P (mg kg‒1)
Plant height (cm)
W (% FC)
P (mg kg‒1)
Stem diameter (mm)
W (% FC)
P (mg kg‒1)
d
e
f
Root length (cm)
W (% FC)
P (mg kg‒1)
Stem diameter (mm)
N (mg kg‒1)
P (mg kg‒1)
Root length (cm)
N (mg kg‒1)
P (mg kg‒1)
g
h
i
R/S ratio
W (% FC)
N (mg kg‒1)
Dry biomass (g plant‒1)
N (mg kg‒1)
P (mg kg‒1)
R/S ratio
N (mg kg‒1)
P (mg kg‒1)
