# Supplementary material for: Improvement of growth performance of Amorpha fruticosa under contrasting regime of water and fertilizer in coal-contaminated spoils using response surface methodology
Source: BMC Plant Biol. 2020 Apr 25;20:181. doi: 10.1186/s12870-020-02397-1 (PMC7183614; doi:10.1186/s12870-020-02397-1)
Supplement: Supplementary file 2 — Additional file 2: Figure S2. Response surface plots showing the effect of soil-water (W), nitrogen (N) and phosphorus (P) on the leaf water potential (LWP) (a-b), photosynthesis rate (Pn) (c), transpiration rate (Tr) (d-e), stomatal conductance (Gs) (f-g), water use efficiency (WUE) (h-i), chlorophyll a (Chl a) (j-l), chlorophyll b (Chl b) (m-o) and total chlorophyll (Total Chl) content (p). [file 12870_2020_2397_MOESM2_ESM.pptx]

## Slide 1
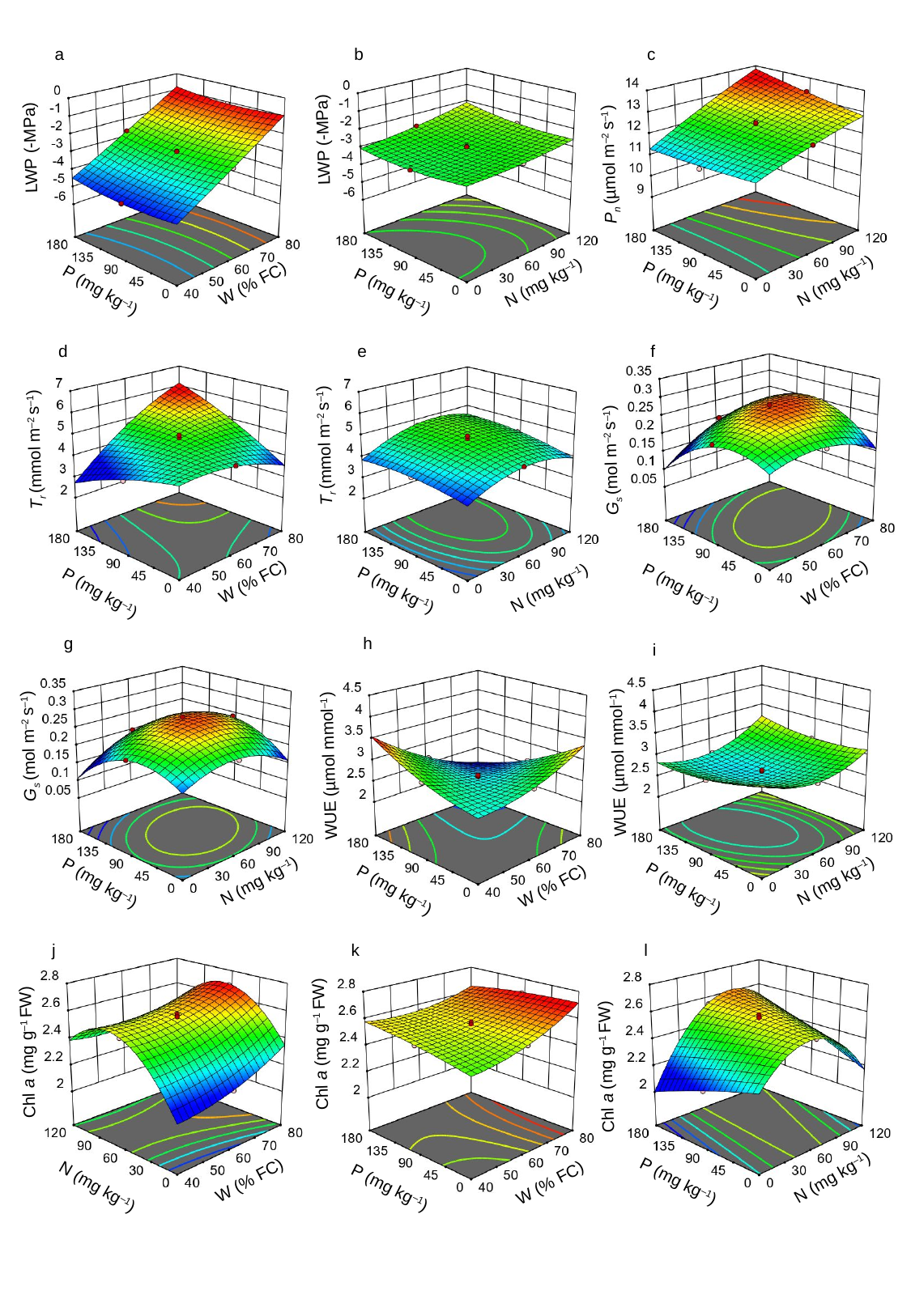

a
b
c
LWP (-MPa)
N (mg kg‒1)
P (mg kg‒1)
LWP (-MPa)
W (% FC)
P (mg kg‒1)
Pn (µmol m‒2 s‒1)
N (mg kg‒1)
P (mg kg‒1)
d
e
f
Gs (mol m‒2 s‒1)
W (% FC)
P (mg kg‒1)
Tr (mmol m‒2 s‒1)
N (mg kg‒1)
P (mg kg‒1)
Tr (mmol m‒2 s‒1)
W (% FC)
P (mg kg‒1)
Gs (mol m‒2 s‒1)
N (mg kg‒1)
P (mg kg‒1)
g
h
WUE (µmol mmol‒1)
W (% FC)
P (mg kg‒1)
i
WUE (µmol mmol‒1)
N (mg kg‒1)
P (mg kg‒1)
j
k
l
Chl a (mg g‒1 FW)
W (% FC)
P (mg kg‒1)
Chl a (mg g‒1 FW)
W (% FC)
N (mg kg‒1)
Chl a (mg g‒1 FW)
N (mg kg‒1)
P (mg kg‒1)

## Slide 2
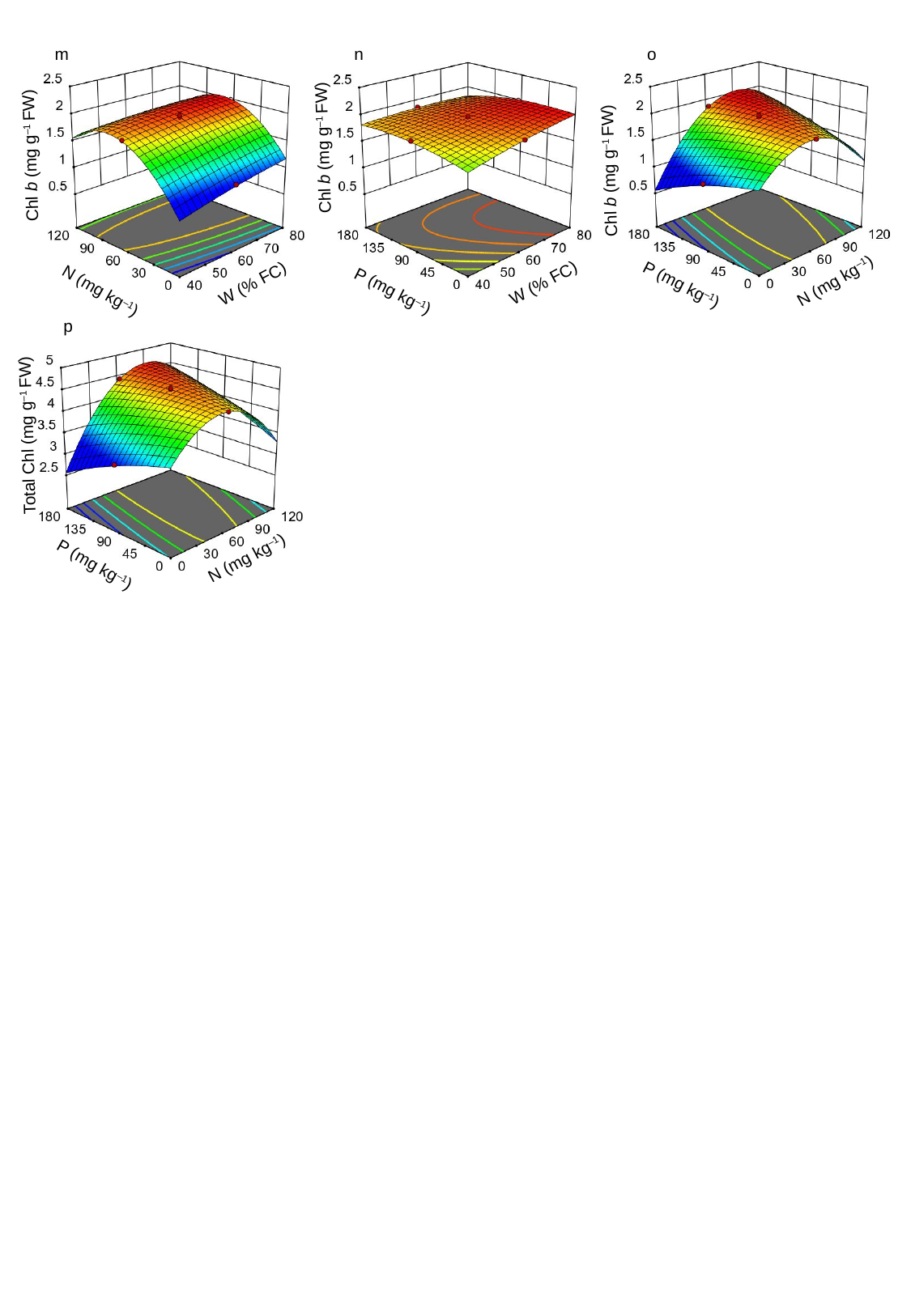

m
n
o
Chl b (mg g‒1 FW)
W (% FC)
P (mg kg‒1)
Chl b (mg g‒1 FW)
W (% FC)
N (mg kg‒1)
Chl b (mg g‒1 FW)
N (mg kg‒1)
P (mg kg‒1)
p
Total Chl (mg g‒1 FW)
N (mg kg‒1)
P (mg kg‒1)
