# Supplementary material for: Improvement of growth performance of Amorpha fruticosa under contrasting regime of water and fertilizer in coal-contaminated spoils using response surface methodology
Source: BMC Plant Biol. 2020 Apr 25;20:181. doi: 10.1186/s12870-020-02397-1 (PMC7183614; doi:10.1186/s12870-020-02397-1)
Supplement: Supplementary file 3 — Additional file 3: Figure S3. Response surface plots showing the effect of soil-water (W), nitrogen (N) and phosphorus (P) on the malondialdehyde (MDA) (a) and proline (Pro) (b-c) contents, and activities of superoxide dismutase (SOD) (d-e), catalase (CAT) (f-g), and peroxidase (POD) (h-i). [file 12870_2020_2397_MOESM3_ESM.pptx]

## Slide 1
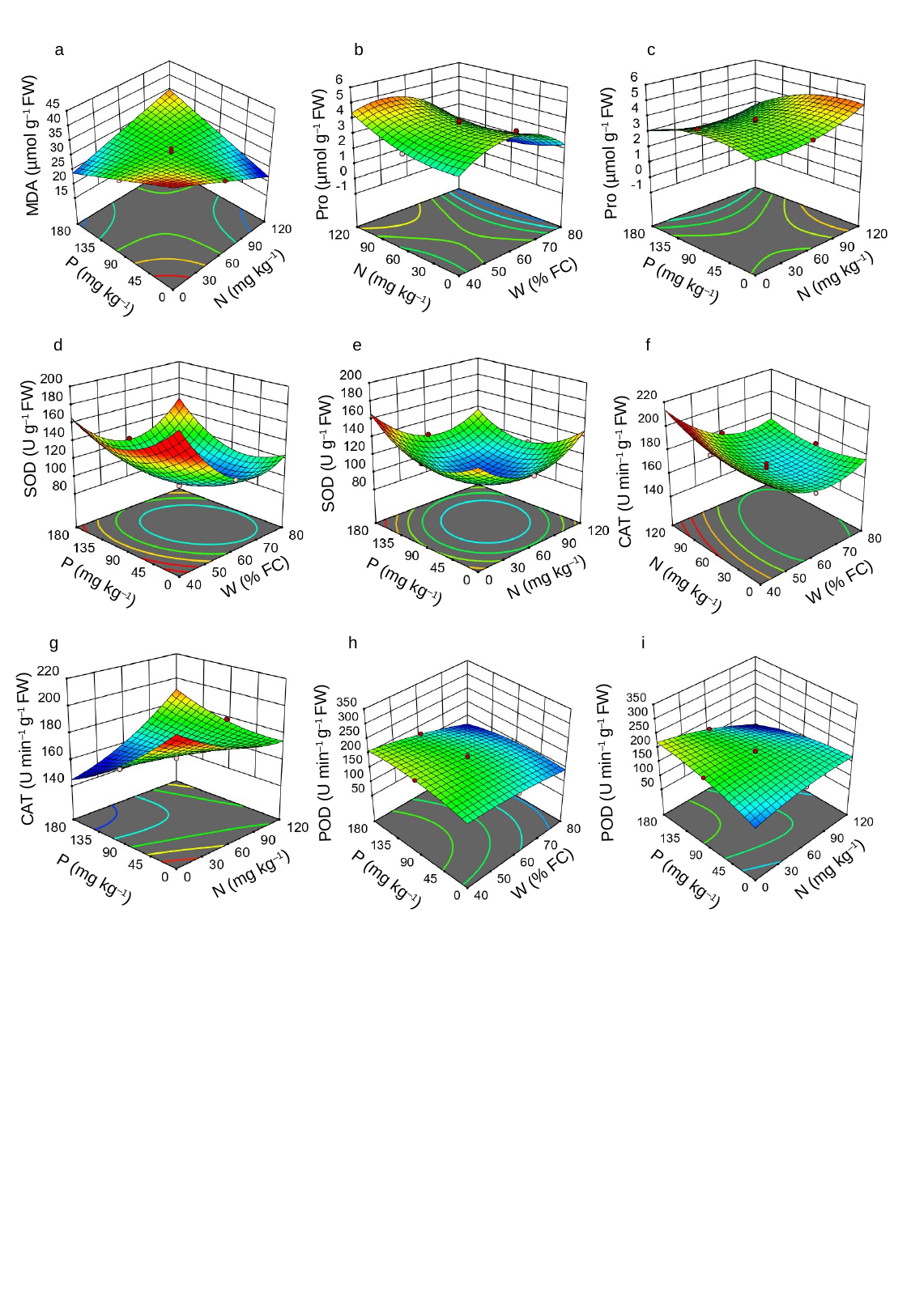

a
b
c
MDA (µmol g‒1 FW)
N (mg kg‒1)
P (mg kg‒1)
Pro (µmol g‒1 FW)
N (mg kg‒1)
P (mg kg‒1)
Pro (µmol g‒1 FW)
W (% FC)
N (mg kg‒1)
d
e
f
SOD (U g‒1 FW)
W (% FC)
P (mg kg‒1)
SOD (U g‒1 FW)
N (mg kg‒1)
P (mg kg‒1)
CAT (U min‒1 g‒1 FW)
W (% FC)
N (mg kg‒1)
g
h
i
CAT (U min‒1 g‒1 FW)
N (mg kg‒1)
P (mg kg‒1)
POD (U min‒1 g‒1 FW)
W (% FC)
P (mg kg‒1)
POD (U min‒1 g‒1 FW)
N (mg kg‒1)
P (mg kg‒1)
